# Reflective Practices to Study Group Dynamics: Implement Empowerment and Understand the Functioning of Groups

**DOI:** 10.3389/fpsyg.2021.786754

**Published:** 2021-11-29

**Authors:** Nadia Rania, Ilaria Coppola, Laura Pinna

**Affiliations:** Department of Education Sciences, School of Social Sciences, University of Genoa, Genoa, Italy

**Keywords:** reflective practices, qualitative methods, groups dynamics, group reflections, Italy

## Abstract

**Introduction:** Individual and group reflection practices are qualitative methods useful in a group context to develop group skills and more awareness of the dynamics that take place within the group to which one belongs.

**Aim:** The aim of this work is to highlight how individual reflective practices and group reflections contribute to the development of group skills. More specifically, the effectiveness of relevant group dynamics is investigated, with the aim of creating a space for reflection, and activation for individual and group empowerment.

**Participants:** The participants were 130 university students (86% female), resided in northwestern Italy, and had a mean age of 27.5 years (*SD* = 7.52). They were randomly divided into 23 groups (ranged from four to nine members).

**Method:** The participants engaged in several online training activities and at the end of every activity they completed individual reflection practice in which they presented both positive and negative aspects related to the group experiences. Then they participated in online group reflections that allowed them to reflect on the group dynamics, particular attention given to factors such as climate, participation and roles played by each participant in a variety of training activities. This study analyzes 130 individual reflective practices and 23 group reflections. The data collected through individual reflective practices and the transcripts of the group reflections were analyzed using grounded theory. Two independent judges analyzed and categorized the data and then identified the main common categories that emerged by the support of Nvivo software.

**Results:** From the analysis of the content, it is determined that the participants, based on the construction of the individual and group reflections, paid greater attention to the dynamics that occurred within the group during the various activities in which they participated, thus allowing them to be more aware of the various factors that affected the importance attributed to the different roles, the climate, and their active participation.

**Conclusion:** Combined, these factors allowed participants to strengthen their relationships with each other and enhance the cohesion of the group.

## Introduction

The individual and group reflective practices are a qualitative tool that in a group context allows to analyze how the members develop more awareness and group skills that favor the implementation of individual and group empowerment. Regarding “group skills,” literature means those essential characteristics that a group must have for its proper functioning such as communication, leadership, creativity, organization, and collaboration (Tarricone and Luca, 2002). Reflective practices are defined by some authors (Schön, 1993; Perrenoud, 1994; Pescheux, 2007; Nuzzaci, 2011) as the ability to make inferences, consciously using previous experiences, theorizing the practice through a formalization of knowledge of action. In particular, the literature has highlighted those reflective practices are considered a qualitative tool useful in groups. The group has also been defined as a learning tool (Bruno and Dell’Aversana, 2018), a place par excellence that favors learning methods and complex training, as each member of the group brings different skills and qualities that are comprehended through comparison, discussion and conflict during the construction of an articulated competence (Pojaghi, 2005). Small group work, in particular, facilitates social well-being, social connection and self-motivation, all of which influence the development of professional growth (Dari et al., 2021). The group-work methodology is an experiential learning tool that initiates social and cultural change processes that can be implemented throughout professional fields, such as school, social, health, work, etc. (Bolocan Parisi et al., 1988). In general, the objectives of a working group include the achievement of a sharing of the issues discussed, a common purpose and a mutual commitment to action. The role of the facilitator is fundamental not so much for the content treated by the group but for maintaining the structure of the group and the success of the process. Groups have their own emotional life that influences and is influenced by their members, as each participant experiences the pressure of achieving what they want and balancing what is good for the group (Phillips and Phillips, 1993). Small group work, also referred to as cooperative learning, is a methodology that involves small groups of individuals working together on a common task (Villalba, 2007; Steen et al., 2008, 2014). As an example, teamwork is an important component of school curricula (Paisley and Milsom, 2007), and the participation of young university students in such teamwork is crucial for career development, especially as it is linked to higher academic achievement (Thanh et al., 2008; White, 2011). This methodology also improves peer interactions (Akos et al., 2007) and is comprised of processes like those encounters in the workplace. For example, teamwork in school requires many of the same skills as teamwork in the work environment, such as effective communication, brainstorming, collaboration, trust building, and conflict resolution (Tindale and Kameda, 2000). Group settings offer students the opportunity to observe and develop new skills through peer interactions and allow a safe and therapeutic space to practice those skills (Perusse et al., 2009). This allows them to gain strength and confidence, which carries over to other aspects of their lives, while also enhancing their advocacy skills (Diemer et al., 2010; Dari et al., 2021). Finally, as supported by Vinatier (2006), working and talking with others about lived experiences allows one to process and understand the facts and events experienced and to thereby formulate interpretations that are useful as one reconsiders certain practices.

The members of the group, reflect on and compare themselves within the contexts of their professional practice and question the sense of self in practice as they develop their own professional identity (Bruno and Bracco, 2016). Reflective practices concern reflective doing or operating, referring to the complex of modalities, strategies, procedures concretely implemented in reflection. For Perrenoud (2001) in particular, reflective practice is connected with reflexivity, as an identity that measures reality in nature or in the consequences of reflection on daily actions in one’s professional environment. In fact, such reflective practices are used in different contexts, including the supervision (Heffron et al., 2016) of work and learning, which then promotes the construction of new knowledge that allows for the creation of meaning and new actions, as well as more familiar contexts (Boud et al., 1985; Nuzzaci, 2011).

Reflecting on action allows an analysis developed from experience, a learning based on the “circularity between action and reflection” on which quality also depends (Bruno and Dell’Aversana, 2018, p. 346). Furthermore, reflection improves the quality of practice and service delivery (Heffron et al., 2016). The use of reflective practices is experimented in different contexts such as work and/or training, where the use of group work is a fundamental tool. According to Ghaye (2001), reflective practices allow workers (both as individuals and as groups) to develop empowerment with respect to tensions in the workplace. Empowerment is achieved through reflection, a critical analysis of one’s working reality to transform it and improve individual and working group well-being and satisfaction. Moreover, the group’s reflective practices in social work have also been redefined in online mode following the pandemic: Parrello et al. (2021) report a study on the use of the multi-vision group for the first time used in online mode, reflective group that aims to protect the well-being of workers.

With respect to the use of reflective practices with university students, some studies (Esposito et al., 2017, 2021) have highlighted the importance of using the narrative mediation path in an innovative group counseling method to favor the reflection or the development of the capacity for reflection and mentalization. In addition, reflective writing encourages students to express personal emotions, and through this, the learner has the opportunity to reflect on the entire learning process (Rué et al., 2013), thus giving way to a transformation of learning through a process of metacognition (YuekMing and Abd Manaf, 2014). Parrello et al. (2020) highlight how reflexivity is fundamental for practice in the context of studies related to social educational work.

### Reflective Practice as a Tool of Awareness

In the early 1930s, Dewey (1933) defined reflective practice as looking at the previous experience to draw useful meanings for the next experience, a learning style that Finlayson (2015) describes as a collective, public nature within a learning organization, i.e., an intellectual experience. This definition has been expanded and revised over the years (Marshall, 2019; Greenberger, 2020). Indeed, Schön (1975), building upon the definition of Dewey (1933), defines reflection as a learning strategy useful for professionals seeking to achieve a greater awareness of their implicit knowledge. Although different definitions of reflection have been proffered over the years, there appears to be agreement on the function that it performs. Specifically, reflection contributes to the development of self-awareness and the improvement of the decision-making process (Greenberger, 2020). In the 21st century, educational research has increasingly focused attention on reflection as a fundamental aspect of the learning process (Sabtu et al., 2019). Nuzzaci (2011), referring, for example, to the practice of reflexivity in the school environment, emphasizes that in the process of reflection, interaction with colleagues is fundamental for the implementation of informed actions. Sabtu et al. (2019) argue that reflective practices help students both achieve a greater understanding of the academic materials learned and develop student and learning processes throughout their lives (Finlay, 2008). As reported by YuekMing and Abd Manaf (2014), the writing process itself is a fundamental learning tool in that it offers the possibility for students to become autonomous and competent as they reflect on what they have learned and clarify and process thoughts and knowledge in a coherent and structured way, which leads to a greater understanding of the discipline (Rué et al., 2013). Reflective practice can include both a dimension of solitary introspection and a more defined group dimension, the latter of which implies a critical dialog with others (Finlay, 2008). For example, Bruno and Dell’Aversana (2018) state that teamwork favors the development of reflective learning through the activation of a professional identity and the shared responsibility of students regarding the learning environment. The authors also suggest that the group has been recognized as a powerful training tool, within which, based on their reflections and on the comparisons of the contexts of their professional practices, learners develop their professional identities by questioning their sense of self (Bruno and Bracco, 2016). Accordingly, in reflective learning environments, as students are active and responsible agents of their own learning, evaluation is not focused on the expected results but on the process of acquiring knowledge and the development of interpersonal and communication skills (Boud et al., 1985; Bruno and Dell’Aversana, 2018).

### The Group Dimension of Reflective Practice

From the studies in the literature, it emerges that the group dimension, compared to the individual dimension, is a more problematic approach than is a reflective approach. According to Bion (1961), who introduces the rational working group model, the rationality of the group, albeit stronger than that of the individual, is in a constant struggle with emotional tendencies that push toward objectives that are in conflict with those explicitly shared by the group. There are studies (Bondioli, 2015; Savio, 2016) of educational teams in childcare services regarding their participatory and reflective capacities with respect to group dimension. In this regard, a training model of promotion from within is proposed that addresses the obstacles that may impede the reflective and participatory capacities of the group, such as apprehension in situations of learning from experience, lack of definitiveness with respect to the correct path, or the individual need to belong to the group, which may result in one becoming lost in the group.

According to Mezirow (2003), reflexivity is linked to the meaning that an individual attributes to his or learning by interpreting the experience and generating new knowledge *via* the sharing of the skills possessed. Other authors (Wenger et al., 2002) underscore that the working groups are real “communities of practices.” That is, they are a group of people who together, within a defined space, deepen knowledge and experience by sharing the same themes, passions, and similar problems. When these “communities of practices” share the same professional contexts, a shared reflexivity to act by bringing together experiences, histories, cultures, and common languages is created to build shared solutions for problematic situations (Albanese et al., 2010).

There are studies that focus on reflective practices in teacher training (Calderhead, 1989; Parsons and Stephenson, 2005; Black and Plowright, 2010; Crotti, 2017) and studies that emphasize reflective group supervision in the workplace (Proctor, 2008; Knight et al., 2010), which is regarded as a process that offers opportunities to strengthen reflective capacity within and among the professionals involved. In this respect, Finlay (2008) highlights how reflective practice is used in supervisory contexts through the presence of the dimension of dialog. From another perspective, group reflective supervision is used in health care as a tool to develop the reflective capacity of staff (Heffron et al., 2016). Collin and Karsenti (2011) accentuate that from a theoretical point of view, the literature has focused more on the individual perspective of reflective practice, showing little interest in the collective dimension, which is explicitly evident when there is the presence of another and an verbal interaction ensues, which is an intrinsic characteristic. Furthermore, the authors highlight the importance of interpersonal (group activities), intrapersonal (reflective writing), and online (written messages and reactive feedback) dimensions within what they define as interactive reflective practice.

## Aims

The aim of this work is to highlight how individual reflective practices and group reflection contribute to the development of group skills. The effectiveness of relevant group dynamics is investigated, with the aim to create a space for reflection, discussion, and implementation, evaluating their effects with respect to how the tool of reflection of the group is used by students.

## Participants

The participants are 130 students (86% females and 14% males), lived in northwestern Italy and had a mean age of 27.5 years (22–56 years, *SD* = 7.52). Furthermore, they enrolled in the master’s degree courses of the Department of Social Sciences (Psychology and Educational Sciences). The participants already had experience of online groups, as, in the midst of an emergency crisis caused by the COVID-19, the educational courses, in which the students had taken part in the previous semester, had been organized and conducted on the Teams platform.

## Materials and Methods

Each participant at the end of the activities proposed weekly during the course wrote an individual reflective practice in the form of a diary structured by thematic areas, highlighting the positive and negative aspects of the group experiences, their involvement in the various activities, what worked or not in the group and the reactions of the participants to the empowerment experience. Group reflection practices were then carried out within the group, together with the researcher, which allowed to reflect on the group dynamics created during the various training activities, focusing on the most relevant ones, with particular attention to factors such as the climate, participation and the role played by each one. Each group reflection produced a collective written practice. In both modalities, individual reflective practice and group reflective practice, there were no limits of space, everyone was free to follow their own narrative modalities in the individual level while in the group dimension there was a group negotiation on how much to produce in the group narrative.

## Procedure

Participants joined the study on a voluntary basis with formal enrollment in a group training course, held during October and November 2020. The participants were randomly divided into 23 groups of 4–9 members. The students participated in several online training activities using the Teams platform, according to previously established rules: all participants had to have their screen on, they spoke one at a time. Groups were self-managed and there was no formal role of conductor. Each group completed a group work assigned by the teacher linked to the theoretical contents explained previously (for example designing a structured role playing, analyzing a film segment from the point of view of leadership styles and roles that emerged, designing a focus group). At the end of each activity proposed by the teacher, each participant in the group filled out an individual reflective practice and at the end of the course each group filled out a collective reflective practice after a group discussion, managed in this case by the teacher. There was no minimum or maximum writing limit for these practices. Only the research group had access to both individual and group reflective practices. The researchers assigned each individual reflective practices an individual code (IPP1 = Individual Practice Participant 1, IPP2, etc.) and a group code for collective practice (CPG1 = Collective Practice Group 1, CPG2, etc.).

## Data Analysis

The data collected through individual reflective practices and the transcripts of the group reflection on the several activities were analyzed through grounded theory (Glaser and Strauss, 1967). Two independent researchers analyzed the qualitative data following the constant comparison analysis technique (Leech and Onwuegbuzie, 2011). The approach is based on grounded theory (Glaser and Strauss, 1967) and is supported using the software Nvivo12 (2018). The grounded theory, through a systematic and flexible methodology, helps to build models based on empirical data. The software was used to create a graphic representation and define a model based on the previously identified categories. The model is composed of nodes and sub-nodes, and their labels represent the categories and sub-categories identified by the researchers that exemplify the main themes that emerged.

## Results

This study analyzed 130 individual reflective practices and 23 group reflective practices. The total body of text individual reflective practices was 413,821 words, while that relating to group ones was 18,238 words. The total textual corpus was 432,059 words.

From the analysis of the practices, some sub-categories have been identified for each category, some that unite both types of practices, others specifically referring to individual practices and finally others still only referring to collective ones. For each subcategory, some significant sentences that best represent the subcategory itself are reported.

An analysis of the content indicates that the participants, due to the construction of individual and group reflections, paid greater attention to the dynamics that occurred within the group during the various activities. This enhanced their awareness of the various factors that affect the importance attributed to the different roles, the climate, and the degree of active participation. Combined, these factors allowed them to get to know each other better and strengthened the cohesion of the group.

**FIGURE 1:**
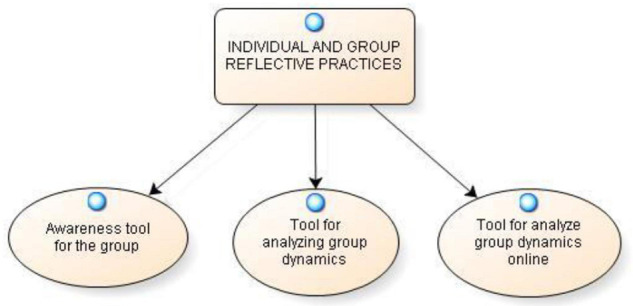
Categories of individual and group reflective practices.

In particular, there are some interesting aspects that emerged from the analysis of reflective practices, and which constitute the three categories identified by the researchers, that are represented in the model shown in Figure 1.

The first category refers to the use of reflective practice as an **awareness tool for the group**. The second category identified reflects on how individual and group reflective practices have been a **tool for analyzing the dynamics of the group**. Through the drafting of reflective practices, the participants identified salient points relating to the dynamics that occurred during the performance of group activities, touching both positive and critical aspects. A final aspect to consider is the way the activities were conducted. In fact, individual reflective practices proved to be a **tool for analyze group dynamics online**, from which some critical issues emerged, but also unexpectedly positive implications.

### Awareness Tool for the Group

**FIGURE 2:**
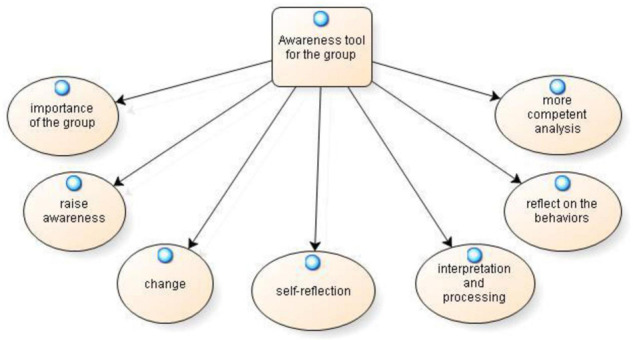
Awareness tool for the group.

From the analysis of individual and collective reflective practices, it emerged the category **awareness tool for the group**, within which seven sub-categories have been identified: importance of the group, raise awareness, change, self-reflection, interpretation, and processing, reflect on the behaviors and more competent analysis as shown in Figure 2.

The first subcategory, tool that brings out the importance of the group, emerges from both individual reflective practices “*In general, from all the reflective practices, I have reached the awareness of the importance of the contribution of the group in soliciting in each of us new food for thought that, alone, we would not even take into consideration*” (IPP51), that of group “*Reflecting in a general way on group activities, we hypothesized that they were characterized by positive interdependence and individual responsibility […] this allowed the increase of cognitive and social skills. We helped each other, and we were co-responsible; we established our rhythm of work; and we corrected each other” (CPG23)*.

The analysis of individual reflective practices, however, found that these specific practices were a valid tool in **raise awareness** as to what occurred within the group: “*The technique of reflective practice allowed me to clarify what had happened within the group and become aware of what I had done well and what I still needed to improve on. Putting this in writing helped me a lot, compared to the simple reflection that I could only have done in my mind at the end of the experience” (IPP105)*. Another participant also stated, “*The reflective practice activity carried out led me to various ideas about our ways of working in groups” (IPP20)*. Others viewed the individual reflective practices as a tool for **change:** “*I therefore tried to involve others and call other’s names for the interventions, recalling what was already written in the last reflective practice and wanting to develop and take on a different role more often than in the previous group experience” (IPP65)*.

Individual reflective practice is also seen as a **self-reflection** tool within the group: “*It can be concluded that this experience was very useful as an introspective practice that led us to reason about ourselves” (IPP88)*. *“This writing practice, on the other hand, made me realize that I could be more proactive during group activities*” (IPP40). Some participants noted that individual reflective practice is also an **interpretation and processing** tool: “*The atmosphere that characterizes the group activity is one of mutual understanding*…*During the experience, as far as I am concerned, a wide spectrum of conflicting feelings and reactions followed one another. For this reason, I decided to carry out the first reflective practice on this activity, focusing further on the interpretation and elaboration of the same” (IPP12)*.

Furthermore, the group reflective practices suggest that some participants felt the reflective practices allowed them to **reflect on the behaviors** of those in the group. “…*deeper reflection of our behaviors within the group and those of others” (CPG4)*. Finally, the participants noted that group reflective practices allowed the group to perform a **more competent analysis** of the training contents and behaviors identified within the group. *“Through reflective practices, we were able to look at the contents learned in class and our socio-relational behaviors with more analytical and competent eyes” (CPG18)*.

### Tool for Analyzing the Dynamics of the Group

**FIGURE 3:**
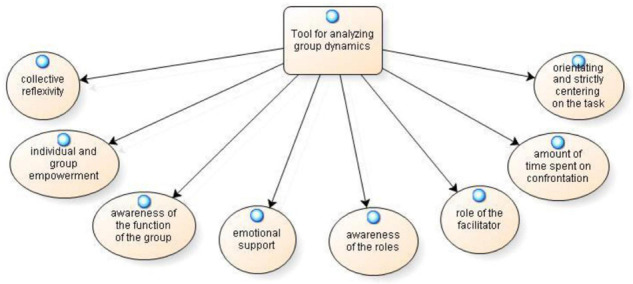
Tool for analyzing group dynamics.

As reported in Figure 3, from the analysis of textual corpus emerged the category **tool for analyzing the dynamics of the group**, consisting of eight sub-categories: collective reflexivity, individual and group empowerment, awareness of the function of the group, emotional support, awareness of the roles, role of the facilitator, amount of time spent on confrontation and orientating and strictly centering on the task.

As can be seen from the sub-categories reported, the group and the dynamics that occur within it have been the object of reflection in the various reflective practices analyzed. Particularly, from individual and group reflective practices, it is clear that group work allows for the development of a **collective reflexivity:** *“During the group work I perceived a collective reflection on the issue addressed” (IPP111). “The conversation stimulated dialog and reflection; the group gave rise to a productive confrontation; and each component introduced new themes, which were exposed in a climate of openness and non-judgmental listening” (CPG1)*. It was further noted that there was a willingness to listen to all members. “*All group members were willing to listen and participate in order to build a common reflection*” (CPG123). The drafting of individual and collective reflective practices also led the participants to focus their attention on those factors that allow, through the activation of **individual and group empowerment**, the achievement of a pre-established group goal:

“*For example, now I am aware that in a group there are people with different roles and that beyond their role they can help the group both to improve relationships and to achieve goals” (IPP28).*

*“Group activity first of all promotes personal and group empowerment but also the development of expressive and communication skills, stimulating creativity” (CPG15).*

“*At the group level, there was a collaborative, participatory climate of considerable attention*” (CPG10).

*“A highly motivated group can work well to achieve the ultimate goal and, consequently, a sense of well-being and gratification can arise for all members of the group” (CPG17).*

An analysis of the individual writing process determined that the participants have one greater **awareness of the function of the group**. Specifically, reflection through practice:“…*allowed me to understand how the group works, what are the characteristics of the way others behave*… *and it allowed me to act in subsequent meetings with serenity*” (IPP99) One of the functions attributed to the group is relative to **emotional support**, which is evident in one participant’s reflective practice: “*This experience made me understand the real potential of the group in the emotional sphere; it was impactful and very illustrative of the power a group can have in supportive areas” (IPP128).*

Furthermore, the collective writing process indicates that group activities, important from a training and relational point of view, are useful in acquiring greater **awareness of the roles** within the groups. *“This experience was enriching not only from a training point of view but also from a relational point of view, and it allowed us to understand and have greater awareness of the roles assumed” (CPG5)*.

Finally, the drafting of the group reflective practice is a critical **role of the facilitator** who led the group and shared creative and thoughtful comments. “*The intervention of the facilitator was important […] he was able to ask questions that generated very creative and thoughtful comments” (CPG22)*.

However, in addition to the positive aspects related to being part of a group, there was no lack of critical perspectives in the individual reflective practices. Concerns addressed by the participants included the **amount of time spent on confrontation**, both of which represented obstacles to achieving the task: “*The fact that each of us always has observations to make and, more generally, what we say can also be a critical issue. All time that had to be given to others meant we had to deal with a lot, and we risked not being able to finish the job*” (IPP33). The analysis also revealed how some participants suffered from **orientating and strictly centering on the task**, thus causing them to not pay attention to others: “*In my opinion, a certain attention toward others has failed. We have focused more on always being ‘on the piece’ from the point of view of the objective of the task, leaving out the possible point of view of others, which I believe has instead been listened to only as a function of his possible contribution useful for the purposes of the task, but without leaving real space for the person” (IPP75).*

### Tool to Analyze Group Dynamics Online

**FIGURE 4:**
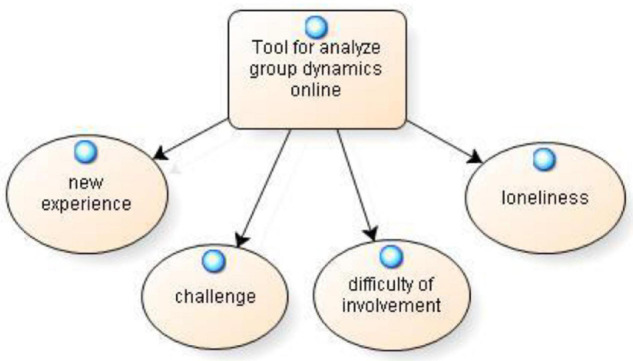
Tool for analyze group dynamics online.

The category tool to **analyze group dynamics online** it refers to how individual and group reflective practices proved to be a tool to bring out and reflect on the group dynamics that took place online. Particularly, four sub-categories have been identified: new experience, challenge, difficulty of involvement, and loneliness (see Figure 4).

As regard **new experience** many participants claimed as “*My perception of the group has partly changed from previous experiences because I am experimenting with a new form of online interaction with mostly unknown people*. *However, what remains unchanged compared to direct collaboration is the sense of mutual commitment. It is thanks to the cohesion of the group that the work will proceed in the best way since we are motivated not only by the activity but also by the social action proposal being drawn up” (IPP44)*. From the reflective practices, it also emerged that this experience was perceived as **a challenge** that had to be overcome. “Group *work that is done in an online mode seemed to be something unattainable, but it turned out to be a real challenge that I managed to overcome” (IPP112)*. Finally, individual reflective practices gave rise to concerns about **difficulty of involvement** the group as involvement was hindered by telematics. At an individual level, emphasis is placed on difficulties associated with emotional involvement: “*I think if we had had the chance to discuss in person, the process would have been even more emotionally engaging” (IPP43)*. Another participant stated, “*technology has allowed us to create groups at a distance, but it does not allow us to recover that warmth given by the relationships in presence” (IPP95)*. On a collective level, however, the perceived **loneliness** behind the screen is emphasized and is credited with making interactions among members difficult. “*Instead, the number of people who feel even more alone behind the screen has increased, thus increasing even more the discomfort for those who speak or expose themselves in front of strangers was almost manageable. In these conditions we notice a distinct difficulty in interaction that can be perceived as little participation because of the way we perceive the other changes, whereas in person we notice the non-verbal behaviors that still express a message” (CPG12)*.

## Discussion

This study reports the findings of both individual and group reflective practices, with regard to the analysis of dynamics that occur within a group and the challenges confronting online groups. From the analysis of a textual corpus, it emerges that these practices are experienced as tools that accentuate the importance of the group’s ability to develop new reflections on an individual level and increase cognitive and social skills on a collective level. Furthermore, data highlights how both individual and collective practices are a tool that facilitates not only greater **awareness of group dynamics** but it also facilitates **change** thanks to the memory of the writing of a previous reflective practice, they had implemented a different behavior and role within the group. As reported by Mezirow (2003), the process of acquiring awareness may promote change. More specifically, the experience is translated, through reflection, into a changed conceptual perspective that forces the education professional and his community of belonging toward continuous identity crises and the loss of dogmatic temptations. That said, awareness comes from the action of the subject and the representation that he has created for himself (Nuzzaci, 2011).

The research highlights the use of reflective practice as a tool for **self-reflection** within the group and for **the interpretation and processing of activities**, and as such, it is fundamental for the group itself. The literature (Boud et al., 1993) gives credence to the importance of the group as a powerful training tool, particularly in the context of team and problem-based learning where learning is socially and culturally constructed and influenced by the socioemotional setting in which it occurs. In particular, the collective reflective practices, on the other hand, allow the group **to reflect on its behaviors**, thereby activating a more **competent analysis** of the contents learned and the behaviors implemented and providing **greater awareness of the roles** embraced within the group.

More specifically, an analysis of the results indicates that both individual and collective reflections are capable of leading to a greater awareness of the functioning and importance of the group as well as to the development of **collective reflexivity and individual and group empowerment**. Both from individual and collective practices, it became clear that a collaborative climate, the presence of different roles and high levels of motivation are fundamental to group success; as claimed, in fact, by Tarricone and Luca (2002), organization and collaboration are fundamental pillars of group skills. The literature (Fonagy and Target, 2005) speaks of reflective functioning as the process by which the behavior of others is understood, interpreted, and given meaning on the basis of thoughts, feelings, beliefs, and desires that motivate that behavior. From an analysis of the results, it is concluded that individual reflective practices are a qualitative tool that allow people who are part of a group to activate a **process of introspection, interpretation, and functional elaboration** of the group itself. Infact, in many case the writing is dictated by having experienced a wide range of conflicting feelings and reactions throughout the group activity. Furthermore, it should be emphasized that the writing of group reflective practices has allowed us to highlight that group activities increase cognitive and social skills and that, within the group itself, there is positive interdependence and co-responsibility in achieving the group’s shared goal/s. In this regard, a review of Høyrup and Elkjaer (2006) indicates that from an individualized perspective, collective reflection is perceived as an individual cognitive learning process that occurs in social contexts. Again, the role of conversation is fundamental and is defined as a dimension that stimulates dialog and reflexivity (Collin and Karsenti, 2011). Reflection on action also includes the notion of reflection on the relationship, that is, on how to create and maintain links with others and on the dynamics of groups and organizations (Nuzzaci, 2011). Individual reflective practices have also highlighted certain criticalities that working in a group entails a dimension that has not emerged in collective reflective practices. In fact, it is highlighted that, while on the one hand, circular communication can lead to an increase in interactions between participants, and thus favor comparison (Rania et al., 2021), on the other hand, there is the risk that discussion times will be increased, **thereby risking the ability to fully achieve the task**. Consistent with this, Brown and Miller (2000) underscored that the pressure of time to achieve results can be a source of stress. Other participants stressed that the activity of the group **strictly focused on the task** can lead to a lack of attention toward its members or, in general, toward the individual perspectives of the other group members. Bavelas (1950) and Leavitt (1951) proposed a model for describing communication networks and how they affect the productivity of the group. More specifically, the members of a group are conceived as beings in a relationship joined through the bonds of communication, where distance and centrality are quantitative indices to describe the different types of networks. According to the authors, centralized groups complete their tasks more easily than those groups arranged in a decentralized way. However, the latter are more satisfied with the work performed. Brown and Miller (2000) differentiate the types of communication based on the complexity of the task, showing that groups working on simpler tasks tend to use more centralized communication modes compared to groups engaged in more complex tasks. Finally, the participants, through their individual and group reflections, identified the particularities of the relational dynamics of groups that meet only through online platforms. It is, in fact, a new form of online interaction, which is increasingly widespread due to the COVID-19 pandemic and which, as highlighted in the literature, allows the individual to have positive experiences that enhance social and relational skills (Saldanha, 2020; Rania et al., 2021). In fact, interacting with unknown people in a virtual environment naturally resulted in change. However, the participants explained that the collaboration and group commitment to achieve their goals remained unchanged. In this regard, it has been found that the construction of a reflective group constitutes a protective factor for the well-being of workers (Parrello et al., 2021). The implementation of the group through the online mode was experienced by some participants as a critical issue, as there were, at least initially, difficulties linked to both emotional involvement and personal interactions, particularly for those who experience challenges speaking and interacting with strangers. The literature further suggests that the use of online platforms can present challenges, for example, with respect to non-verbal communication (Rania et al., 2021).

## Conclusion

The analyzed results highlight that individual and collective reflective practices foster a greater awareness of the knowledge learned (Sabtu et al., 2019), the dynamics that occur within the group, and the emotions experienced, all of which, together, provide the opportunity to reflect on the learning process in its complexity (Rué et al., 2013). Furthermore, the writing of reflective practices compels students to accept co-responsibility in the learning context, which is fundamental in the development of meaningful and reflective learning (Bruno and Dell’Aversana, 2018). Among the limits of the research, it is evident that the participants would be students of the humanistic-social area, therefore more inclined, due to the type of training, to reflect on relational aspects and group dynamics. It would be interesting to use this type of reflective practices in different training contexts to verify how they can become tools of individual awareness also in other contexts and can allow the analysis of group dynamics even with subjects with scientific training.

Despite the limits highlighted, this research has also underlined some strengths; in fact, brought out as learning from experience, through reflective practices and using the group as a training tool, albeit online, can be an effective approach when used in a variety of contexts. For example, its use can stimulate reflection and comparison with one’s own context, questioning both oneself in practice and one’s own identity (Bruno and Bracco, 2016).

Among the implications related to this study, we highlight its usefulness in training professionals who work in teams or even for groups of operators who use the online mode in the workplace. as well as for professions that work mainly online. As Parrello et al. (2021) point out reflective group work can be an organizational tool to maintain the well-being of workers, even more useful during the lockdown period linked to COVID-19.

Finally, although group reflective practices have received little attention (Collin and Karsenti, 2011), this research allows us to affirm that the writing of these practices allows the participants to become more aware of their roles during the learning process in a group context that favors the development of both individual and group empowerment.

## Data Availability Statement

The datasets presented in this article are not readily available because the data cannot be shared for ethical reasons related to privacy, but the authors undertake to make the data available if requested for a valid reason. Requests to access the datasets should be directed to NR, nadia.rania@unige.it.

## Ethics Statement

Ethical review and approval was not required for the study on human participants in accordance with the local legislation and institutional requirements. However, the research was carried out in accordance with the Ethics Research Recommendations of the Italian Association of Psychology and in accordance with the Declaration of Helsinki. Participation was entirely voluntary, confidential and anonymous. The participants were informed that they were free to withdraw from the study at any time. The patients/participants provided their written informed consent to participate in this study.

## Author Contributions

NR conceived the original idea of the study and supervised the results of this work. NR, IC, and LP contributed to the data processing and analysis, and wrote and organized the manuscript, presented the methodology, procedure and the data section. All authors discussed the results, presented the conclusion, and reviewed the manuscript and approved the final version for submission.

## Conflict of Interest

The authors declare that the research was conducted in the absence of any commercial or financial relationships that could be construed as a potential conflict of interest.

## Publisher’s Note

All claims expressed in this article are solely those of the authors and do not necessarily represent those of their affiliated organizations, or those of the publisher, the editors and the reviewers. Any product that may be evaluated in this article, or claim that may be made by its manufacturer, is not guaranteed or endorsed by the publisher.

## References

[B1] Akos P. Hamm J. Mack S. Dunaway M. (2007). Utilizing the developmental influence of peers in middle school groups. *J. Spec. Group Work* 32 51–60. 10.1080/01933920600977648

[B2] Albanese O. Businaro N. Fiorilli C. Zorzi F. (2010). “Rischi e risorse nel contesto scolastico per la professione insegnante (Risks and resources in the school context for the teaching profession),” in *La Scuola Come Contesto (School as a Context) Prospettive Psicologico-Culturali*, eds Ligorio B. Pontecorvo C. (Roma: Carocci), 215–224.

[B3] Bavelas A. (1950). Communication patterns in task-oriented groups. *J. Acoust. Soc. Am.* 22 725–730. 10.1121/1.1906679

[B4] Bion W. R. (1961). *Experiences in Groups and Other Papers.* London: Tavistock Publication, 10.4324/9780203359075

[B5] Black P. E. Plowright D. (2010). A multi-dimensional model of reflective learning for professional development. *Reflect. Pract.* 11 245–258. 10.1111/j.1365-2923.2010.03722.x 20716096

[B6] Bolocan Parisi L. G. Gervasio Carbonaro G. Viciani Bennici A. (1988). *Il lavoro di Gruppo. Metodologie, Tecniche, Formazione, Aggiornamento Dell’operatore (Group Work. Methods, Techniques, Training, Operator Updating).* Roma: Carocci Faber.

[B7] Bondioli A. (2015). “Promuovere dall’interno: un’estensione dell’approccio del “valutare, riflettere, restituire (Promote from within: an extension of the “evaluate, reflect, give back),” in *La Valutazione di Contesto nei Servizi per L’infanzia Italiani (Context Assessment in Italian Childcare Services). Riflessioni ed Esperienze*, eds Bondioli A. Savio D. (Bergamo: Edizioni Junior), 41–56.

[B8] Boud D. Cohen R. Walker D. (1993). *Using Experience for Learning.* Buckingham: Open University Press.

[B9] Boud D. Keogh R. Walker D. (eds) (1985). *Reflection: Turning Experience into Learning.* London: Kogan Page.

[B10] Brown T. M. Miller C. E. (2000). Communication networks in task-performing groups: effects of task complexity, time pressure, and interpersonal dominance. *Small Group Res.* 31 131–157. 10.1177/104649640003100201

[B11] Bruno A. Bracco F. (2016). Promoting safety through well-being: an experience in healthcare. *Front. Psychol.* 7:1208. 10.3389/fpsyg.2016.01208 27570515 PMC4981592

[B12] Bruno A. Dell’Aversana G. (2018). Reflective practicum in higher education: the influence of the learning environment on the quality of learning. *Assess. Eval. High. Educ.* 43 345–358. 10.1080/02602938.2017.1344823

[B13] Calderhead J. (1989). Reflective teaching and teacher education. *Teach. Teach. Educ.* 1 43–51. 10.1016/0742-051x(89)90018-8

[B14] Collin S. Karsenti T. (2011). The collective dimension of reflective practice: the how and why. *Reflect. Pract.* 12 569–581. 10.1080/14623943.2011.590346

[B15] Crotti M. (2017). La riflessività nella formazione alla professione docente (Reflexivity in the formation of the teaching profession). *Edetania Estud. Y Propuestas Soc.* 59 85–106.

[B16] Dari T. Chan C. D. Del Re J. (2021). Integrating culturally responsive group work in schools to foster the development of career aspirations among marginalized youth. *J. Spec. Group Work* 46 75–89. 10.1080/01933922.2020.1856255

[B17] Dewey J. (1933). *How We Think: a Restatement of the Relation of Reflective Thinking to the Educative Process.* Boston, MA: D.C. Heath & Co Publishers.

[B18] Diemer M. A. Wang Q. Smith A. V. (2010). Vocational interests and prospective college majors among youth of color in poverty. *J. C. Assess.* 18 97–110. 10.1177/1069072709350906

[B19] Esposito G. Karterud S. Freda M. F. (2021). Mentalizing underachievement in group counseling: analyzing the relationship between members’ reflective functioning and counselors’ interventions. *Psychol. Serv.* 18 73–83. 10.1037/ser0000350 30932505

[B20] Esposito G. Ribeiro A. P. Gonçalves M. M. Freda M. F. (2017). Mirroring in group counseling: analyzing narrative innovations. *Small Group Res.* 48 391–419. 10.1177/1046496417697149

[B21] Finlay L. (2008). Reflecting on ‘Reflective practice’. *Paper Presented at the Practice-Based Professional Learning Paper 52*, (Milton Keynes: The Open University).

[B22] Finlayson A. (2015). Reflective practice: has it really changed over time? *Reflect. Pract.* 16 717–730. 10.1080/14623943.2015.1095723

[B23] Fonagy P. Target M. (2005). Bridging the transmission gap: an end to an important mystery of attachment research? *Attach. Hum. Dev.* 7 333–343. 10.1080/14616730500269278 16210243

[B24] Ghaye T. (2001). “Empowerment through reflection: competence for the new millennium or a case of the emperor’s new clothes?,” in *International Perspectives on Competence in the Workplace*, ed. Velde C. (Dordrecht: Springer), 10.1007/978-94-010-0742-9_11

[B25] Glaser B. G. Strauss A. L. (1967). *The Discovery of Grounded Theory: Strategies for Qualitative Research*, 1st Edn, Chicago, IL: Aldine de Gruyter.

[B26] Greenberger S. W. (2020). Creating a guide for reflective practice: applying Dewey’s reflective thinking to document faculty scholarly engagement. *Reflect. Pract.* 21 458–472. 10.1080/14623943.2020.1773422

[B27] Heffron M. C. Reynolds D. Talbot B. (2016). Reflecting together: reflective functioning as a focus for deepening group supervision. *Infant. Ment. Health J.* 37 628–639. 10.1002/imhj.21608 27783848

[B28] Høyrup S. Elkjaer B. (2006). “Reflection – taking it beyond the individual,” in *Productive Reflection at Work: Learning for Changing Organizations*, eds Boud D. Cressey P. Docherty P. (New York, NY: Routledge), 29–43.

[B29] Knight K. Sperlinger D. Maltby M. (2010). Exploring the personal and professional impact of reflective practice groups: a survey of 18 cohorts from a UK clinical psychology training course. *Clin. Psychol. Psychother.* 17 427–437. 10.1002/cpp.660 19937716

[B30] Leavitt H. J. (1951). Some effects of certain communication patterns on group performance. *J. Abnorm. Psychol.* 46 38–50. 10.1037/h0057189 14813886

[B31] Leech N. L. Onwuegbuzie A. J. (2011). Beyond constant comparison qualitative data analysis: using NVivo. *Sch. Psychol. Q.* 26 86–97. 10.1037/a0022711

[B32] Marshall T. (2019). The concept of reflection: a systematic review and thematic synthesis across professional contexts. *Reflect. Pract.* 20 396–415. 10.1080/14623943.2019.1622520

[B33] Mezirow J. (2003). *Apprendimento e Trasformazione: Il Significato Dell’esperienza e il Valore Della Riflessione Nell’apprendimento Degli Adulti.* Milano: Raffaello Cortina.

[B34] Nuzzaci A. (2011). Pratiche riflessive, riflessività e insegnamento. *Stud. Educ.* 12 9–27.

[B35] Paisley P. O. Milsom A. (2007). Group work as an essential contribution to transforming school counseling. *J. Spec. Group Work* 32 9–109. 10.1080/01933920600977465

[B36] Parrello S. Fenizia E. Gentile R. Iorio I. Sartini C. Sommantico M. (2021). Supporting team reflexivity during the COVID-19 lockdown: a qualitative study of multi-vision groups in-person and online. *Front. Psychol.* 12:719403. 10.3389/fpsyg.2021.719403 34421770 PMC8377588

[B37] Parrello S. Iorio I. De Rosa B. Sommantico M. (2020). Socio-educational work in at-risk contexts and professional reflexivity: the multi-vision group of “Maestri di Strada”. *Soc. Work Educ.* 39 584–598. 10.1080/02615479.2019.1651260

[B38] Perrenoud P. (1994). *La formation des Enseignants Entre Théorie et Pratique.* Paris: L’Harmattan.

[B39] Perrenoud P. (2001). *Développer la Pratique Réflexive Dans le Métier D’enseignant.* *Professionnalisation et Raison Pédagogique.* Paris: ESF.

[B40] Parsons M. Stephenson M. (2005). Developing reflective practice in student teachers: collaboration and critical partnerships. *Teach. Teach.* 11 95–116.

[B41] Perusse R. Goodnough G. E. Lee V. V. (2009). Group counseling in the schools. *Psychol. Sch.* 46 225–231. 10.1002/pits.20369

[B42] Pescheux M. (2007). *Analyse des Pratiques Enseignantes en FLE/S. Mémento Pour une Ergonomie Didactique du FLE.* Paris: L’Harmattan.

[B43] Phillips L. D. Phillips M. C. (1993). Faciliated work groups: theory and practice. *J. Oper. Res. Soc.* 44 533–549. 10.2307/2584511

[B44] Pojaghi B. (2005). *Il Gruppo Come Strumento di Formazione Complessa. Il Farsi e il Disfarsi Delle idee.* Milan: FrancoAngeli.

[B45] Proctor B. (2008). *Group Supervision: A Guide to Creative Practice*, 2nd Edn, London: Sage.

[B46] Rania N. Coppola I. Pinna L. (2021). Adapting qualitative methods during the COVID-19 era: factors to consider for successful use of online photovoice. *Qual. Rep.* 26 2711–2729. 10.46743/2160-3715/2021.4863

[B47] Rué J. Font A. Cebrián G. (2013). Towards high-quality reflective learning amongst law undergraduate students: analysing students’ reflective journals during a problem-based learning course. *J. Qual. Assur. High. Educ.* 19 191–209. 10.1080/13538322.2013.802575

[B48] Sabtu N. I. Matzin R. Jawawi R. Jaidin J. H. (2019). Enhancing critical reflection in higher education. *AIP Conf. Proc.* 2138:050026. 10.1063/1.5121131

[B49] Saldanha K. (2020). A view from the other side: a senior’s view of participating in online groups during the pandemic. *Soc. Work Groups* 1–6. 10.1080/01609513.2020.1848331

[B50] Savio D. (2016). *Formare Équipe Riflessive e Participative Nei Servizi Educative Per L’infanzia.* Paris: RELAdEI, Formation del Profesorado de Education Infantil.

[B51] Schön D. (1993). *Il Professionista Riflessivo. Per Una Nuova Epistemologia Della Pratica Professionale.* Bari: Dedalo.

[B52] Schön D. A. (1975). Deutero-learning in organisations: learning for increased effectiveness. *Organ. Dyn.* 4 2–16.

[B53] Steen S. Bauman S. Smith J. (2008). The preparation of professional school counselors for group work. *J. Spec. Group Work* 33 253–269. 10.1080/n01933920802196120

[B54] Steen S. Henfield M. S. Booker B. (2014). The achieving success everyday group counseling model: implications for professional school counselors. *J. Spec. Group Work* 39 29–46. 10.1080/01933922.2013.861886

[B55] Tarricone P. Luca J. (2002). “Successful teamwork: A case study,” in *Quality Conversations, Proceedings of the 25th HERDSA Annual Conference*, Perth, WA, 640.

[B56] Thanh P. T. H. Gillies R. Renshaw P. (2008). Cooperative learning (CL) and academic achievement of Asian students: a true story. *Int. Educ. Stud.* 1 82–88. 10.5539/ies.v1n3p82

[B57] Tindale R. S. Kameda T. (2000). “Social sharedness” as a unifying theme for information processing in groups. *Group Process. Interg.* 3:123. 10.1177/1368430200003002002

[B58] Villalba J. A. (2007). Incorporating wellness into group work in elementary schools. *J. Spec. Group Work* 32 31–109. 10.1080/01933920600977556

[B59] Vinatier I. (2006). “Des dispositifs de co-explicitation: un travail de conceptualization de son activité par l’enseignant, le formateur,” in *Proceedings of the Faciliter les Apprentissages Autonomes, 7ème Colloque Européen sur L’autoformation*, (Toulouse-Auzeville: ENFA), 18–20.

[B60] Wenger E. Mc Dermott R. Snyder W. (2002). *Cultivating Communities of Practice.* Boston, MA: Harvard Business School Press.

[B61] White R. (2011). A sociocultural understanding of mediated learning, peer cooperation and emotional well-being. *Emot. Behav. Diffic.* 16 15–33. 10.1080/13632752.2011.545600

[B62] YuekMing H. Abd Manaf L. A. (2014). Assessing learning outcomes through students’ reflective thinking. *Procedia Soc. Behav.* 152 973–977. 10.1016/J.SBSPRO.2014.09.352

